# Rabies and Pinnipeds Reviewed: Premonitions, Perturbations, and Projections?

**DOI:** 10.3390/vetsci13020200

**Published:** 2026-02-19

**Authors:** Charles E. Rupprecht, Aniruddha V. Belsare

**Affiliations:** 1 College of Veterinary Medicine, Auburn University, Auburn, AL 36849, USA; abelsare@auburn.edu; 2 College of Forestry, Wildlife, and Environment, Auburn University, Auburn, AL 36849, USA

**Keywords:** disease, epizootiology, lyssavirus, mesocarnivores, pinnipeds, rabies, seals, serology, surveillance, vaccination

## Abstract

Rabies is an acute, progressive, viral encephalitis. Warm-blooded vertebrates are susceptible. Major reservoirs reside in the Chiroptera and Carnivora. Among the latter, representatives include dogs, ferret badgers, foxes, jackals, mongooses, raccoons, and skunks. Within the Carnivora, pinnipeds represent a diverse group of >30 extant species. These marine mammals range from the Arctic to Antarctica, but there is no review about rabies in this group. Apparently, only a single 1980 case of rabies occurred from Svalbard in a ringed seal (*Phoca hispida*). However, in 2024, incidental cases appeared within South African Cape fur seals (*Arctocephalus pusillus*). Retrospective testing of archival material identified suspect cases dating back to 2022. Currently, more than 80 cases have been documented in seals. Moreover, a new 2025 focus appeared in Namibia and cases in Angola are predictable. Viral characterization supports spillover infection via rabid black-backed jackals (*Lupulella mesomelas*). A host shift appears likely, with ongoing seal intraspecific transmission. Given the unique nature of this epizootic, implications for the southern hemisphere abound. Unfortunately, comprehensive data are lacking on pinniped specimens examined outside of southern Africa. For example, although Antarctica is considered ‘rabies-free’, minimal international standards for support are unmet. No routine laboratory-based surveillance occurs. This enzootic rabies focus among seals in southern Africa presents unique challenges for the region and a rare opportunity for considering broader surveillance. Besides targeted parenteral vaccination of fur seals, local engagement involves vagrant species, including elephant (*Mirounga leonina*) and leopard seals (*Hydrurga leptonyx*). The void of regional pinniped surveillance, especially encompassing the Southern Ocean would require considerable proactive local resolution and much wider collaboration regarding future concerns to both public health and conservation biology.

## Simple Summary

Rabies is a deadly viral disease that can infect all mammals. However, despite obvious host breadth, some major groups are lacking in rudimentary surveillance. For example, among marine mammals, throughout the 20th century, only a single rabies case was reported in a seal from Norway. Thereafter, a unique rabies outbreak was detected in South African Cape fur seals during 2024, believed to be associated with viral introduction via rabid jackals. A subsequent viral host shift and seal-to-seal transmission appears likely. Besides local public health concerns to residents and coastal tourists, the larger implications include involvement of other seal species elsewhere and potential spread throughout the Southern Ocean, threatening the conservation biology of wildlife in Antarctica, the only supposedly ‘rabies-free’ continent. Proactive surveillance is necessary to develop relevant management plans before broader disease emergence occurs.

## 1. Introduction

Rabies is a socio-politically neglected, broadly distributed, high-consequence, low-prevalence, fatal disease, with continued scientific and public interest in the pathogen and its management in myriad hosts, as reflected in the professional literature and media accounts [1,2,3,4,5,6,7,8,9,10,11,12,13,14,15,16,17,18,19,20,21,22,23,24,25,26,27,28,29,30,31,32,33,34,35,36,37,38,39,40,41,42,43,44,45,46,47,48,49,50,51,52,53,54,55,56,57,58,59,60,61,62,63,64,65,66,67,68,69,70,71,72,73,74,75,76,77,78,79,80,81,82,83,84,85,86,87,88,89,90,91,92,93,94,95,96,97,98,99,100,101,102,103,104,105,106,107,108,109,110,111,112,113,114,115,116,117,118,119,120,121,122,123,124,125,126,127,128,129,130,131,132,133,134,135,136,137,138,139,140,141,142,143,144,145,146,147,148,149,150,151]. This global zoonosis is characterized as an acute, progressive encephalitis of warm-blooded vertebrates, with an extremely variable incubation period [4,5]. Highly neurotropic, etiological agents belong to the Family Rhabdoviridae, Genus *Lyssavirus* [6]. With more than 18 recognized or putative species, *Lyssavirus rabies* (i.e., rabies virus, RABV) is the most important cosmopolitan member, although all lyssaviruses cause the same characteristic disease known as rabies. Domestic dogs are the predominant RABV reservoir responsible for the primary health burden, but the potential wildlife host breadth is huge [7,8,9]. Nevertheless, irrespective of biased surveillance, major viral reservoirs, responsible for independent RABV perpetuation, appear confined to relatively few wild taxa within the Chiroptera and Carnivora, with unpredictable opportunities for viral host shifts [10,11].

Lyssaviruses are dependent upon transit from the CNS to exit portals for transmission. This occurs primarily due to virion-laden saliva via the bite route. Hence, regular conspecific contacts within reservoir populations are the primary determinants for spread and viral maintenance, principally bat-to-bat, dog-to-dog, fox-to-fox, etc. [7,10]. A host switch entails an interspecific transmission event. For example, RABV spillover infections from an infected dog to a human or a rabid fox to livestock represent a routine host switches. Most such cases are epidemiologically dead ends. However, opportunities for limited transmission exist, e.g., if a rabid vampire bat infects a cat which bites a person, or if tissue/organ transplantation proceeds from an unsuspected human case, etc.

As one representative host group, the extant Carnivora consists of more than 275 species (including the domestic dog) [12]. This mammalian order represents a diffuse gradient along a social spectrum [13]. Most terrestrial carnivores are solitary (e.g., ursids) or form small familial groups or packs (e.g., canids). A few may consist of a larger band (e.g., coatis) or more stable sedentary colonies (e.g., meerkats). In addition to well-defined viral reservoirs among red foxes (*Vulpes vulpes*), common raccoons (*Procyon lotor*), and striped skunks (*Mephitis mephitis*), other diverse incidental examples of all these categorial social groupings appear in the rabies literature as natural evidence of opportunity for infection and host switches from reservoir hosts to other Carnivora taxa (Table 1).

Global surveillance and RABV characterization support the concept that routine contact and viral host switching within the Carnivora are common events [14,15,16,17,18,19,20,21,22,23,24,25,26]. In contrast to a host switch, a host shift involves interspecific transmission resulting in viral adaptation and maintenance towards potential perpetuation within the new host (Table 2). By definition, all host shifts begin as host switches. Based upon current evidence, host shifts are much less common than host switches. The genetic or ecological facets that enable viral host shifts are poorly appreciated [11]. Host shifts are not merely frequent indications of individual local examples of concomitant host switches. For example, cases of rabies in Canada among red foxes and skunks in southern Ontario occurred outside the range of Arctic foxes [142,143]. Hence, those cases could not be a multiplicity of individual events sans the existence of the reservoir. Similarly, cases derived originally from canine RABV now perpetuate among mesocarnivores throughout the Americas, despite the long-term elimination of canine rabies (i.e., rabid dogs are no longer responsible for North American cases in foxes, skunks, etc.) [145,147,148]. Additionally, when laboratory introspection via whole-genome and deep sequencing occurs, viral mutations become apparent via phylogenetic analysis, even in the presence of the original parenteral RABV variant in the existing reservoir (i.e., unique host shifts among wildlife in South Africa and elsewhere concomitant with the ongoing perpetuation of incipient canine rabies) [55,123,149,150]. Spatio-temporal overlap, competition, predator–prey interactions, etc., may increase the likelihood of contact, a productive infection, and host switching between species. However, much less commonly, chance events or other factors (e.g., pre- or post-adaptation ecological alterations) may drive positive selection and novel RABV emergence within a new species as an enigmatic host shift.

In contrast to more solitary species (e.g., bears, cougars, weasels, etc.), ‘group’ living may facilitate more frequent contacts and RABV transmission in the face of host switches. No host shifts are documented in less gregarious species. Within the Carnivora, the largest social associations are among the mammalian suborder Pinnipedia (‘flipper-footed’), with 30 extant seal species. Pinnipeds are semiaquatic mammals, exemplified by the walrus (Family Odobenidae, *Odobenus rosmarus*), the eared fur seals and sea lions (Family Otaridae), and the true or earless seals (Family Phocidae), such as the common harbor seal (*Phoca vitulina*), the elephant seals (*Mirounga* spp.), and their relatives. They are found in aquatic environments (typically marine) from the Arctic to Antarctica. As all surveillance is biased, lyssavirus case detection among any mammalian taxa, including pinnipeds, is dependent in part upon human population density, routine monitoring, and the presence of suitable testing laboratories. At the polar margins of Eurasia and North America, human populations are scant, contrasting with the southern extent of pinnipeds along the Australian, South American, and African continents. Seals may congregate along these coasts at sandy, rocky, or ice-covered haul-out sites to rest, molt, breed, and rear young, with diverse life history characteristics, as exemplified by several overlapping southern populations, including vagrants from Antarctica (Table 3).

Seasonally, such pinnipeds can form massive breeding aggregations. Yet, despite their global distribution, unique lifestyles, relative abundance, and opportunities for individual infection via infected terrestrial mammals, relatively little is known about RABV in these marine mammals. This limitation appears somewhat peculiar, considering the commonality of host switches in the Carnivora (Table 1), suspicions about historical pathogen occurrence given observed contact with infected reservoirs, as well as postulated basic mammalian susceptibility and the diversity of other viral pathogens, including adenoviruses, herpesviruses, morbilliviruses, orthomyxoviruses, parapoxviruses, vesiviruses, and others [34,35,36,37,38,39,40,41,42]. Moreover, Antarctica is the only purportedly ‘rabies-free’ continent, regarding the most southern ecological realm. Pinnipeds comprise the representative multi-species resident populations of Carnivora on that ice-covered land mass. Is the contention true that there is no rabies in Antarctica? The objective of this communication was to review the historical literature on rabies in pinnipeds, to describe the current global situation, and to consider the need for contemplating future risks related to pathogen spread, detection, or potential management, given new public health and conservation concerns.

## 2. Tailored Review

To meet our primary objective, we reviewed primary sources of information pertaining to rabies and pinnipeds from ~1789 to 2025. We queried the U.S. National Institutes of Health PubMed database to obtain any relevant citations for historical biomedical literature from MEDLINE, life science journals, online books, and related sources. Search terms included: lyssavirus; marine mammals; pinnipeds; rabies; and seals. In addition, we used a Google search path under ‘news items’ to locate relevant media reports on this topic. Based on the acquired information, we summarized what was known about this specific issue, the limitations to available knowledge, the context to broader reporting and management, available opportunities for any a priori enhanced surveillance based upon relevant diagnostic testing, and the potential implications for broader insights for public health, veterinary medicine, and conservation biology, as described [1,2,3,4,5,6,7,8,9,10,11,12,13,14,15,16,17,18,19,20,21,22,23,24,25,26,27,28,29,30,31,32,33,34,35,36,37,38,39,40,41,42,43,44,45,46,47,48,49,50,51,52,53,54,55,56,57,58,59,60,61,62,63,64,65,66,67,68,69,70,71,72,73,74,75,76,77,78,79,80,81,82,83,84,85,86,87,88,89,90,91,92,93,94,95,96,97,98,99,100,101,102,103,104,105,106,107,108,109,110,111,112,113,114,115,116,117,118,119,120,121,122,123,124,125,126,127,128,129,130,131,132,133,134,135,136,137,138,139,140,141,142,143,144,145,146,147,148,149,150,151].

## 3. Findings

Historical findings were limited. Prior to 2024, only a single case of RABV was reported in a pinniped. This was an incidental finding from Norway [43]. Rabies was diagnosed at Svalbard during 1980 in 12 Arctic foxes (referenced originally as *Alopex lagopus*, but now referred to as *Vulpes lagopus*), three reindeer (*Rangifer tarandus platyrhynchus*), and in one ringed seal (*Phoca hispida*). Subsequently, no RABV antigens were found when brain tissues from 23 polar bears (*Ursus arctos*), 846 arctic foxes, 19 reindeer, and five ringed seals were tested by the direct fluorescent antibody test (DFAT) [44]. In the Arctic, RABV perpetuates in circumpolar fashion due to the Arctic fox, *V. lagopus*, the likely source of the host switch to the seal [45]. No other papers on documented seal cases appeared on this topic in the Arctic or elsewhere throughout the 20th century, including any surveillance in the southern hemisphere. This lack of information on occurrence influenced opinions and actions within the biomedical community throughout the first quarter of the 21st century, related to public health, diagnostic testing, and conservation matters in pinnipeds. Additional insights on this specific topic were primarily dependent upon a series of recent reports from southern Africa.

### 3.1. Basic Prophylaxis Questions

Only a single rabies case was reported in a seal prior to 2024, but public health discussions began earlier, focusing upon risk assessment after human contact [46,47,48,49,50]. Compared to terrestrial carnivores, bites from pinnipeds were uncommon. Most human bites or scratches from seals were treated with wound cleansing, antibiotics (for deep or extensive lesions) and a tetanus booster [46,47,48]. Although a bite from any unavailable wild mammal is considered a potential risk for RABV acquisition, rabies postexposure prophylaxis (PEP) was not or rarely provided after seal bites, either to coastal residents or as a travel medicine consideration for returning tourists [49,50]. In addition to thorough wound washing, modern PEP of transdermal lesions consists of vaccine administration and infiltration of rabies immune globulin into and around all wounds [50,51]. However, prior to 2024, rabies was not considered to be a major epizootiological risk in pinnipeds compared to other viral pathogens. Climate change was raised as one potential factor if impacts to seal ‘haul-out’ frequency on land increased exposure to rabid terrestrial carnivores, such as dogs, foxes, etc. [52]. Understandably, the public health risk after a seal bite was viewed as minimal and PEP was not a routine recommendation [49]. Similarly, rabies pre-exposure prophylaxis (PrEP) was not widely regarded as an occupational necessity for wildlife biologists working with pinnipeds. Given the increasing focus on the causes and frequency of potential exposure incidents by aggressive seals (especially during and after 2019) in some South African and other coastal areas, physicians and public health officials began to raise the basic question of whether PEP should be administered after unprovoked seal bites among swimmers, surfers, and others [46,47,48,49,51].

### 3.2. A Link with South African RABV Dynamics

**Figure 1:**
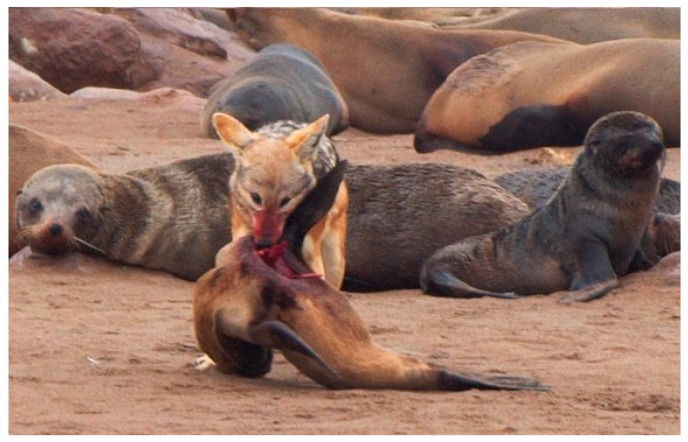
Black-backed jackal (*Lupulella mesomelas*) feeding on a brown fur seal pup in Namibia—route of perceived viral spillover infection from the terrestrial to the marine mammal realm (https://commons.wikimedia.org/wiki/File:An_unwanted_visitor_(cropped).jpg, accessed on 31 October 2025).

The *Journal of Emergency Medicine* review on pinniped bites concluded the ‘… likelihood of rabies is low, and rabies postexposure prophylaxis should be reserved for cases that involve unusually aggressive animal behavior or other factors suggestive of rabies.…’ [48]. Subsequently, during February 2024, a German tourist was bitten while swimming by a South African fur seal and, upon her return to Berlin, received PEP due to the unprovoked aggressiveness of the attack and lack of information or guidelines [51]. Besides animal behavior, one of the factors relevant to PEP consideration is the epidemiological status of the disease where the event occurs [50]. In contrast to the single report in a seal from the Northern Hemisphere, where wildlife rabies perpetuated in the Arctic, perhaps the overall perceived burden of a host switch was lower in those more southern regions that came under renewed scrutiny involving seals bites during 2015–2024? Yet, considering southern Africa, rabies was enzootic throughout the region for over a century [3,8,9,10,19]. Unlike in the Arctic, the disease in Africa was driven by canine rabies, which is responsible for most human fatalities [50]. Additionally, viral spillover infection and host switching to wildlife were common, creating additional sources of exposure to humans and domestic animals [19,20,53,54]. Moreover, after decades of epidemiological introspection, apparent host shifts occurred from the domestic dog to several wild mesocarnivores (Table 2) [55]. Within southern Africa, complex multi-species assemblages of RABV variants perpetuated among domestic dogs, the black-backed jackal (*Lupulella mesomelas*), bat-eared fox (*Otocyon megalotis*), yellow mongoose (*Cynictis penicillata*), and aardwolf (*Proteles cristatus*)—but no mention was made of rabies and seals in this comprehensive 2025 report [56]. Somewhat oddly, based on our literature review, rabies in pinnipeds was not suspected as a major concern, even though regular encounters of potentially rabid canids and Cape fur seals (*Arctocephalus pusillus*) occurred regionally, with opportunities for a host switch (Figure 1).

### 3.3. Nidus Description

The associated index pinniped rabies case in South Africa was not in a fur seal [57,58]. On 20 May 2024, a three-year-old unvaccinated pitbull in the Western Cape in Cape Town was evaluated by a private veterinarian. The dog presented with a fever and a history of fighting with another dog in the household. This pitbull was treated and discharged, but the following day was brought back to the clinic after spending the night attacking furniture in the house. The animal was extremely aggressive on arrival and was euthanized due to a suspicion of rabies, as canine rabies is enzootic within South Africa. However, the owner suspected the dog had been bitten by a Cape fur seal while it was walking on the beach two weeks before the onset of clinical signs. The dog’s brain tested positive for detection of RABV antigens by the DFAT, and was characterized initially as a canid (rather than a mongoose RABV variant or another lyssavirus) biotype by monoclonal antibody typing [57,58]. If the seal exposure was the actual source of infection, the laboratory finding suggested that the seal may have been infected by a rabid dog.

Also, that month, a seal bit two surfers in Muizenberg, a beach town in the Western Cape, prompting warnings to the public to avoid exposures and to report seal bites [57,58,59,60,61]. The attacking seal was captured, died, and a comprehensive postmortem was conducted on 27 May. Preliminary postmortem findings by the local Sea Search Institute researchers and an independent veterinarian revealed that the seal was nutritionally healthy (i.e., weighing 16 kg, with a 13 mm adipose layer). There was no significant neck damage or evidence of blood restriction to the head, so possible effects of strangulation from fouling by fish netting was ruled out. However, significant bruising and severe subdural brain hemorrhage was observed, suggesting that blunt force trauma to the head was a likely cause of death. Such injuries could have been caused by a surfboard collision at the beach. The seal’s brain tested positive for detection of RABV antigens according to an immunohistochemistry (IHC) test [57,58]. As a result of the seal having a rabies diagnosis, the exposed surfers received PEP. Thereafter, the global media took interest in this unfolding situation during May–June of 2024, in what was to become an unprecedented event extending far beyond one infected pitbull, a rabid seal, and a few surfers [60,61,62,63]. Originally, the postulated timeline envisioned an unusual series of events from a suspected rabid dog infecting a seal, with this interspecific case continuing a short chain of transmission with a host switch back from the rabid seal to an unvaccinated dog. The antigenic typing confirmed a canid RABV variant, rather than spillover from a bat lyssavirus (e.g. Duvenhage virus, Lagos bat virus, etc.) or an animal infected with the mongoose viral biotype. Later sequencing of this index case and additional samples revealed identity associated with the black-backed jackal RABV lineage. Greater homology of these rabid seal sequences and subtle differences from the jackal RABV clade supported the contention that a host shift was operational, rather than multiple independent cases of incidental host switches. An outbreak seemed underway by June 2024 [57,58,62,63].

Understandably, rabies was a surprise as a potential overriding explanation for the human attacks from 2019 to 2024. Some were unconvinced that rabies was the explanation. Historically, morbidity, unusual behavior, or aggression in seals varied by species, sex, age, and region, variously ascribed to competition during breeding, maternal defense of newborn pups, extreme crowding, resource depletion, human stressors, organic pollutants, irritation from ectoparasites, marine toxins (e.g., domoic acid), etc. [64,65,66,67,68,69,70,71,72,73,74,75,76,77,78,79,80,81,82,83,84,85]. Given the apparent extreme rarity of the disease in pinnipeds, rabies was not often included in the differential diagnosis list in the evaluation of aggressive, ill, or dead seals. In general, despite multiple surveys for other emerging microbes among pinnipeds, focused surveillance and documentation of rhabdoviruses were lacking [86,87,88,89,90,91,92,93,94,95]. Surveillance for more common and impactful RNA viral pathogens of marine mammals, such as morbilliviruses or highly pathogenic avian influenza, as well as interventional methods for possible mitigation of such agents, dominated the conservation discussion. To protect human health, recommendations for PEP were heightened based upon the laboratory confirmation of rabies in the seals [96]. Questions lingered regarding whether this was an isolated event of limited spillover infections or indicative of a longer-term epizootic.

### 3.4. Expanded Surveillance and Viral Characterization

In addition to the history of potential viral transmission to the original Cape Town dog in 2024, and the case involved with the bite incidents with the surfers during May, untested archival samples dated back to at least 2021 or longer.

**Figure 2:**
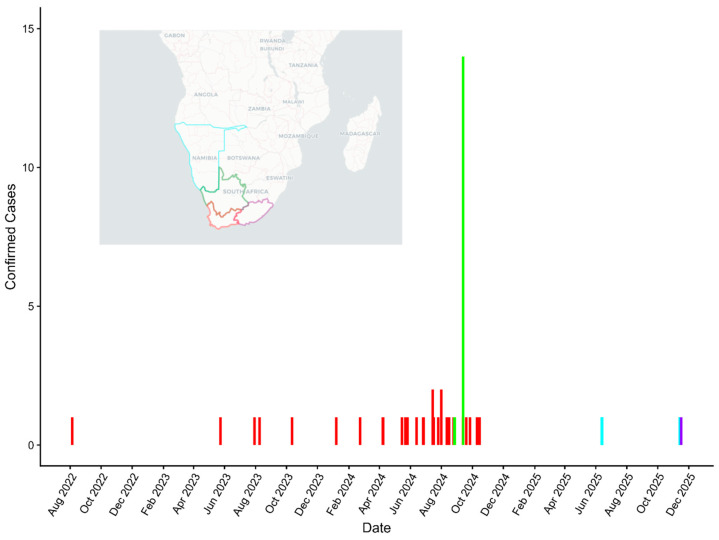
Surveillance reporting for rabid Cape fur seal cases in southern Africa by month, 2022–2025 [57,58,59,97,98,99]. The color of the bars on the graph match case occurrence over time in the areas outlined on the inset map.

Submissions and suspect case testing (retrospectively to 2021 and prospectively thereafter) rose after these initial confirmations in the first fur seals in May and June [57,58,59]. Thereafter, the Agricultural Research Council’s Onderstepoort Veterinary Research group confirmed positive rabies cases in seals from Melkbosstrand on the southwest coast (October 2023), Plettenberg Bay in the Western Cape Province (January 2024), Die Dam near Gansbaai, also in the Western Cape Province (June 2024), and in the Northen Cape province (September 2024). Through September of 2024, of 80 seal brain samples submitted to the laboratory, 36 (45%) tested positive by the DFAT (which was considered the gold standard test). Of 140 formalin-fixed brain samples from August 2022 to 2024, 12 (8.6%) tested positive via an IHC test (which was considered an experimental test, if not validated by testing authorities) [130]. This combination of prospective and archival sample testing revealed a much larger rabies outbreak in time and space (Figure 2). The earliest positive case occurred in August 2022 [151]. Based on submissions, the distribution of rabid seal cases encompassed a wide area. However, a fuller epidemiological appreciation was not possible due to the limitation of available testing results, including all species, age, clinical status, onset dates, locality, denominator data, etc. Moreover, any relationship between rabies cases and seasonal life history (e.g., male seal territory establishment during October and November, with breeding activity during November to December) would be dependent upon more routine monitoring and availability of more than fragmentary case descriptions.

Additional characterization and phylogenetic analysis of RABV obtained from rabid animals at various Cape fur seal colonies in the Western and Northern Cape provinces were reported to display a near 100% sequence identity. Rather than one rabid jackal infecting multiple seals, this observation was supportive of a single conserved source of viral infection, now circulating among multiple Cape fur seal colonies [57,58,59,151]. If these were reflective of individual host switch events only, greater regional diversity would have been apparent. Moreover, an archival RABV sample from a rabid black-backed jackal in Namibia also clustered together with viral sequences from both South African provinces [151]. The phylogenetic analysis supported a theory of introduction from a rabid jackal, followed by seal-to-seal transmission of RABV. Some of the closest-related (98%) seal RABVs were a group from the North-West province, an area representative of the black-backed jackal rabies enzootic focus. Regionally, jackals were known predators within seal colonies (Figure 1). This recognition presented a likely means of contact from rabid jackals to seals. Taken together, such data would implicate introduction via an initial host switch from a rabid black-backed jackal and a representative RABV variant, with subsequent independent maintenance of a host shift event within Cape fur seals (with the potential for infection of other species, as illustrated by the history of the rabid pitbull and attacks upon swimmers during May) [57,58,59]. Enhanced surveillance identified additional South African cases throughout the coastline in 2025, including the first confirmed cases at Jeffery’s Bay in the Eastern Cape during November 2025, spreading from Plettenberg Bay towards Algoa Bay, as well as the first confirmations of rabid seals in Namibia, from Walvis Bay during June, and Pelican Point, also in November [97,98,99,151]. Previously, most of the historical cases in Namibia were associated with rabid dogs in the Northern Communal Areas. Reporting appears linked to the heavy tourist season from November to March due to human–seal interactions. Unfortunately, no comprehensive details on sample sizes, specific gene targets, precise phylogenetic analyses, etc., have appeared in the peer-reviewed literature by 2025, nor in international repositories such as Genbank.

### 3.5. International Engagement and Transdisciplinary Management

Authorities realized the need for rapid, concerted action. With the case confirmation of rabies in seals, the City of Cape Town, in partnership with the Department of Forestry, Fisheries and the Environment, the Two Oceans Aquarium Foundation and Sea Search, convened an expert scientific workshop in July 2024 [100]. Participants included marine mammal experts, veterinarians, marine scientists, government departments and key South African and international stakeholders from Namibia and Australia. Goals were to focus on investigating causes of the increased frequency of unusual and unprovoked Cape Fur Seal bites and aggression, the likelihood of rabies as the causative explanation, discussions about the implications of a wider outbreak, and appropriate management of the Cape Fur Seal population. Several key findings were generated regarding dual public safety and conservation biology themes.

Looking to possible explanations, a recent surge in the number of seals was posited. However, considering local abundance, the Cape Fur Seal population appeared stable. Annual changes fluctuated by 1% to 2%, with most seals on the West Coast of South Africa and Namibia in the largest colonies [100].

Regarding general behavioral changes, a baseline level of aggression in seals was believed to be normal. This arose from multiple causes, including territorial behavior, maternal protection of pups, pain, injury, distress, and poor health associated with various diseases. However, excessive aggression, weakness, and mobility issues, associated with other unusual behavior (e.g., cranial nerve deficits, biting inanimate objects, etc.) was deemed abnormal. It was concluded that domoic acid was not a confounding factor. Levels in local waters were several-fold lower than measured in other regions. While detected in a few seals, concentrations were not believed to be causal for the unusual behavior documented over the past few years. Marine pollution was also not an obvious contributor, as pollution levels in Cape Town did not differ from levels monitored elsewhere along all shorelines. Similarly, overt alterations in predator populations, particularly the absence of certain shark species in the Western Cape, did not easily explain unusually aggressive seals. Rather, rabies was the most likely definitive cause [100].

Moreover, the disease was not simply focal, but appeared to be well-established in the Cape fur seal population [100,151]. By inference from disease investigations in other mesocarnivores, delegates concluded that if a host shift occurred, rabies was unlikely to disappear spontaneously or to be eliminated easily and quickly. Rather, it was considered established, requiring applied One Health management. The long-term effect on the seal population remained unknown. In other cases of rabid Carnivora reservoirs, rabies rose, slowed after peaking, and became enzootic. Although canine rabies was enzootic in southern Africa, all epidemiological and laboratory indications suggested RABV was transmitted to seals from another source and not directly from local domestic dogs [100,151].

Partnerships were deemed critical [100]. A network of coastal authorities and partners needed to implement proactive measures to manage the situation responsibly, regarding case definition, reporting, surveillance, patrolling, euthanasia, necropsy, testing, communications, PEP, animal control, and future research. Ongoing public communication updates on the rabies outbreak were provided through the office of the State Veterinarian. The public was advised to stay clear of coastal wildlife, including seals, particularly as it was illegal to approach, touch, handle, feed, harass or interfere with such wildlife. Persons who encountered seals behaving unusually or aggressively were advised to move away from the animal or leave the water and immediately inform relevant authorities [151]. Prompt medical attention and consultation on the need for PEP was encouraged after seal exposure. Rabies PrEP was an occupational addition for professionals working with seals. Additionally, dog owners were reminded that rabies vaccines had to be up to date, to exercise responsible ownership related to pet supervision, and to prevent their dogs from having contact with seals [100].

For disease control, culling of seals was not recommended compared to vaccination [100]. Nevertheless, with millions of Cape Fur Seals spread from Southern Angola and the Eastern Cape, widespread vaccination of free-ranging seals was not considered possible. However, targeted vaccination was believed to be valuable in some select seals. Although efficacy of rabies vaccination in seals was unknown, there was no reason to expect adverse outcomes. Vaccination recommendations focused on animals that came into regular contact with humans. This included harbor-associated seals and rehabilitation center seals. The Two Oceans Aquarium developed a standard procedure for vaccinating seals, including dosage and administration options. Vaccinated seals were tagged to allow identification and follow up vaccination. Vagrant species, such as visiting elephant seals and Sub-Antarctic fur seals, would be vaccinated as a precautionary measure [101,102]. Such efforts would establish the safety, immunogenicity, logistics, and feasibility of seal vaccination as a responsible precautionary measure to reduce the risk of RABV spread. Vaccinated seals would be tagged when possible to allow longer-term monitoring [100].

Such targeted vaccinations seemed reasonable for a successful individual response, based in part upon extension from other work. For example, newborn harbor seal pups developed high specific RABV antibody responses after immunization with an inactivated rabies vaccine [103]. Additionally, antigen-specific immune responses were measurable after immunization of seals with an inactivated rabies vaccine and/or with tetanus toxoid [104].

The panel’s general considerations over wildlife rabies management to pinnipeds were also based on prior evidence and policy from the field and laboratory, but eschewed certain operational limitations. For over a century, control of rabies in mesocarnivores focused upon lethal population reduction before consideration of more effective, economical, and ethical alternatives, such as parenteral delivery of inactivated commercial RABV vaccine [105]. This tool was used for protection of exotic captive species at risk within zoological exhibits, endangered species in the field, and for free-ranging mesocarnivores in urban and suburban settings. Such trap-vaccinate-and-release (TVR) endeavors were effective, but expensive and limited in scale [105].

The application of TVR was useful in rabies control under certain conditions, but usually as an adjunct to other broader, immunization methods. For example, during the past 60 years, oral rabies vaccination (ORV) evolved from a basic concept to prescribed management [106,107,108,109]. Globally, ORV programs included dogs, foxes, jackals, raccoons, raccoon dogs, and skunks. While there were several different vaccine types that could be maintained within a container for sterility and stability, all were delivered via bait. Why did the panel not support ORV for pinnipeds?

Clearly, the group understood that ORV was not a panacea. Despite the implied meaning in the name Carnivora, many terrestrial mesocarnivores are omnivorous. Exploitation of their dietary breadth, odor cues, food palatability, etc., was extremely useful for ORV distribution in bait. Originally, the focus was on organic sources, such as chicken heads, eggs, and offal, then it switched to commercially produced baits. In contrast, at sea, most pinnipeds consume live prey, such as fish, squid, krill, and other animals (Table 3). Conventional ‘dead’ bait types were unlikely to be consumed. However, the food habits of seals do appear somewhat flexible. Some individuals may resort to scavenging based upon resource availability. In addition, seals may consume fresh dead seafood in captivity. While not immediately applicable to management ad hoc, future field trials with a variety of placebo bait types, seasons, and diel cycles would be useful to discern the likelihood of uptake among fur seals and other pinniped species. Additionally, novel biologics involving new adjuvants and recombinant expression systems or transmissible viral vaccines could be adapted later specifically for pinnipeds for the management of RABV and other infectious diseases [110,111,112,113].

### 3.6. Involvement of Rabies in Pinnipeds Regionally, Including in the Southern Ocean?

**Figure 3:**
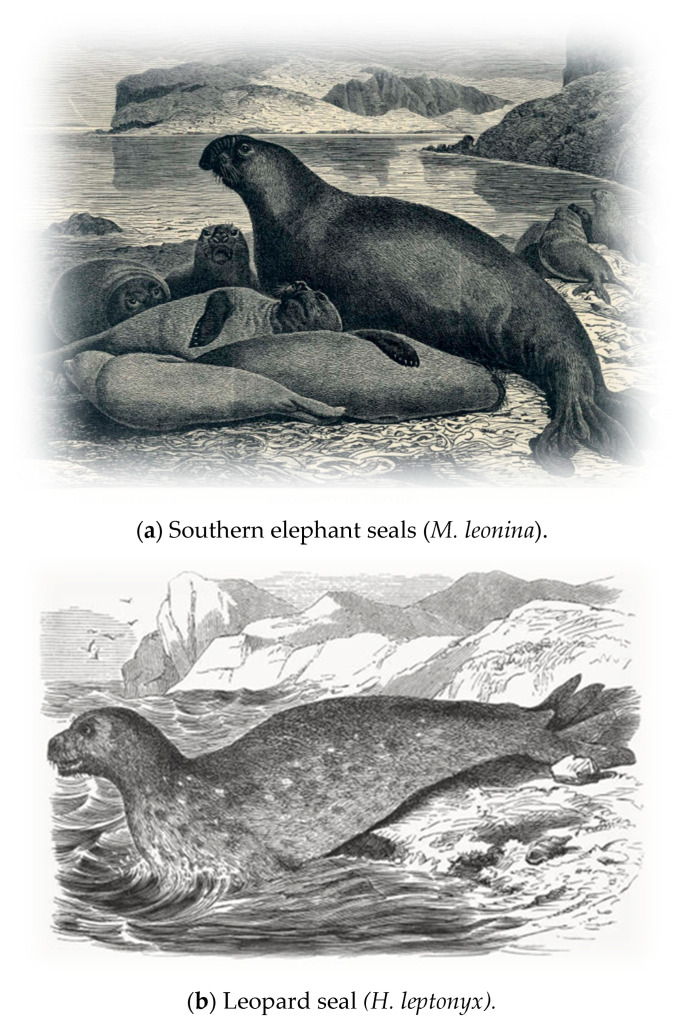
(**a,b**) Potential vagrant pinniped species of interest for viral interspecies infection and spread beyond Africa into the southern oceans. From Ladislaus Weinek (1848–1913)—von Schleinitz: Die Forschungsreise S.M.S. “Gazelle”, 1. Theil. Reisebericht. Mittler Berlin 1889, Tafel 20, Public Domain, https://commons.wikimedia.org/w/index.php?curid=8854641, accessed on 31 October 2025.

**Figure 4:**
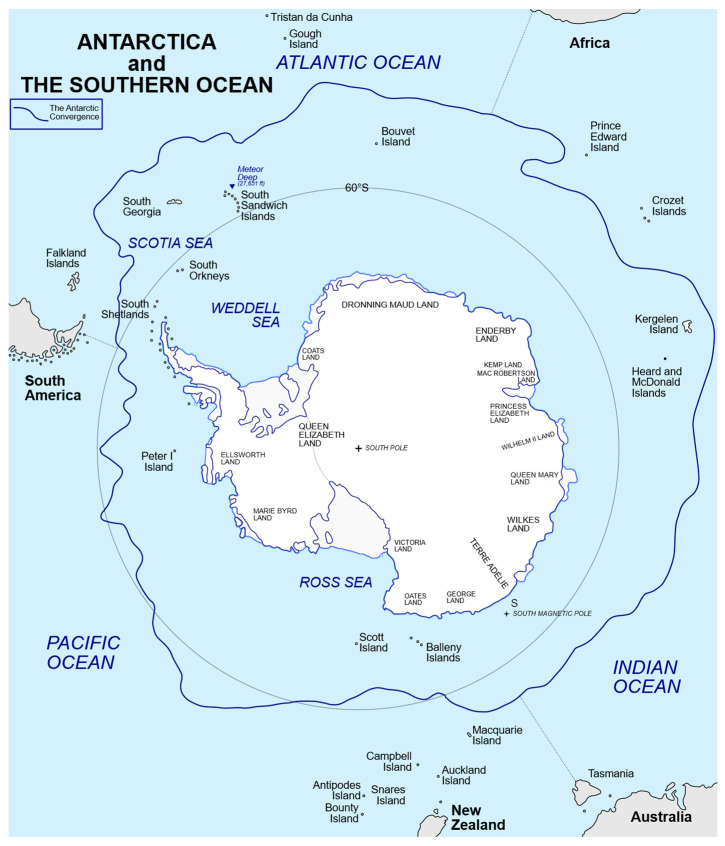
Descriptive map of the Southern Ocean by Hogweard (talk · contribs)—own work based on Antarctic-Convergence-Map. TIF, CC BY-SA 3.0, https://commons.wikimedia.org/w/index.php?curid=38249943, accessed on 31 October 2025.

**Figure 5:**
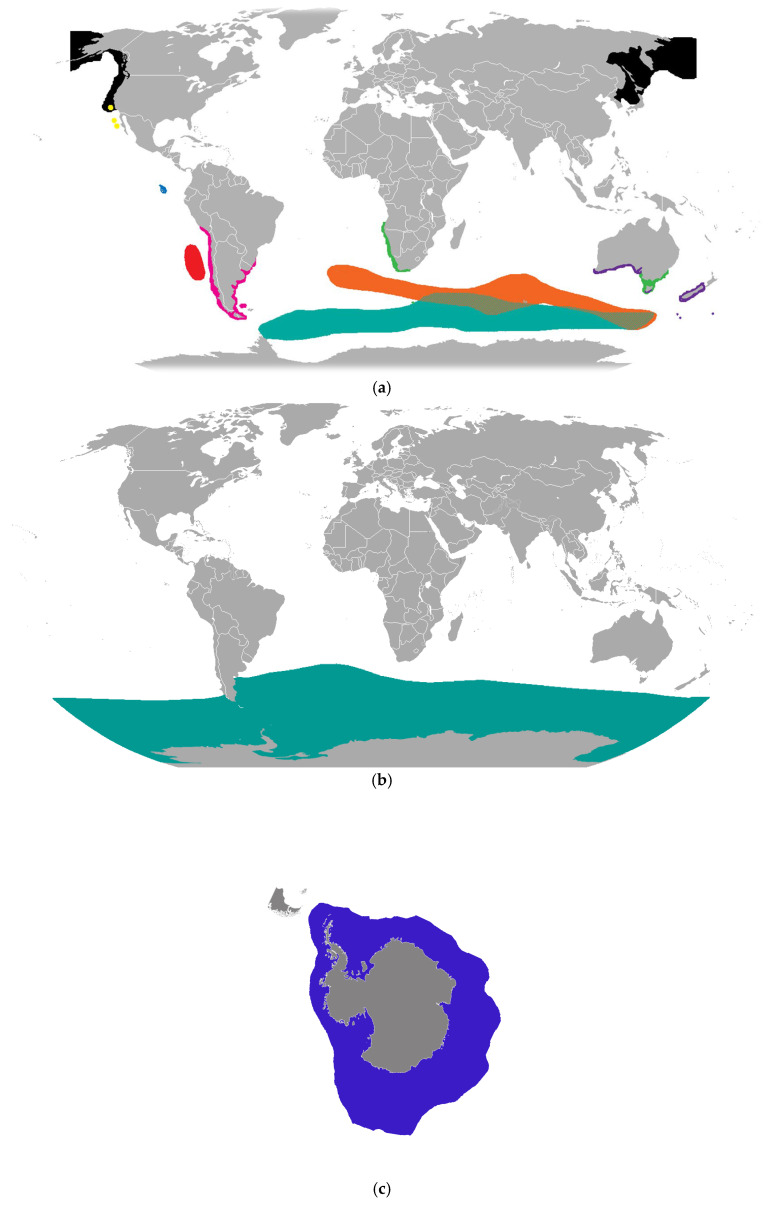

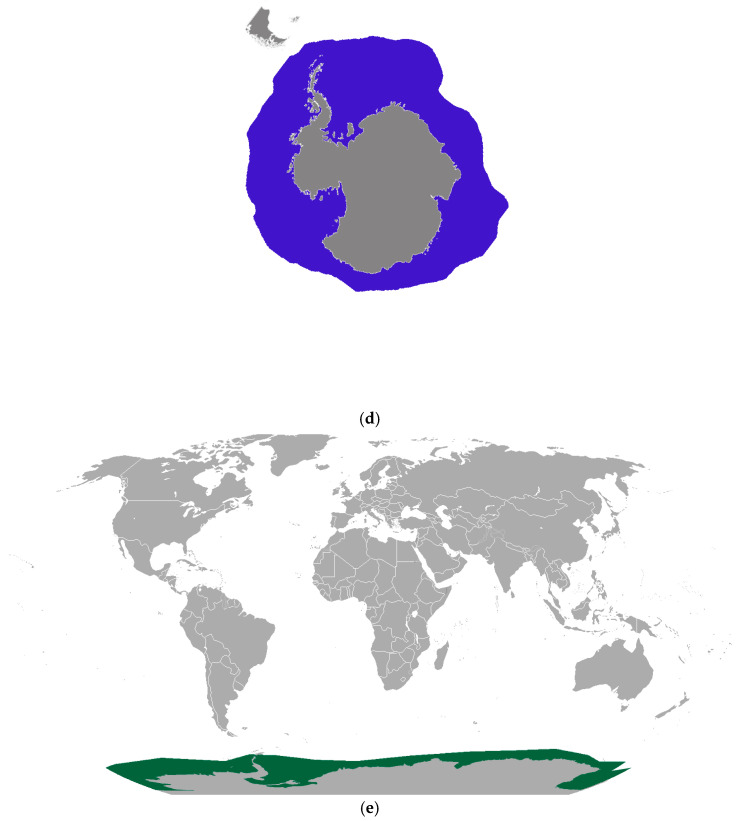
(**a**–**e**) Perceived overlap of selected pinniped species (without designation of potential vagrant species contact), particularly in the Southern Ocean. (**a**) Arctocephalinae taxa: *Arctocephalus pusillus*—green (*A. p. doriferus* is the Australian fur seal subspecies); *Arctocephalus gazella*—cyan; *Arctocephalus townsendi*—yellow; *Arctocephalus philippii*—red *Arctocephalus galapagoensis*—blue *Arctocephalus forsteri*—violet *Arctocephalus tropicalis*—orange; *Arctocephalus australis*—magenta; *Callorhinus ursinus*—black (https://commons.wikimedia.org/wiki/Category:Pinnipedia_distribution_maps#/media/File:Arctophoca.png, accessed on 31 October 2025). (**b**) Southern elephant seal (*Mirounga leonina*) range (Chermundy and IUCN Red List of Threatened Species, https://commons.wikimedia.org/wiki/File:Southern_Elephant_Seal_area.png, accessed on 31 October 2025). (**c**) Distribution of the leopard seal, *Hydrurga leptonyx*, from a south polar projection (By Mirko Thiessen—own image, based on Image: Blank suedpolarregion.jpg, CC BY-SA 2.0 de, https://commons.wikimedia.org/w/index.php?curid=285468, accessed on 31 October 2025). (**d**) Distribution of the crabeater seal, *Lobodon carcinophagus*, from a south polar projection (by Mirko Thiessen—own image, based on http://commons.wikimedia.org/wiki/Image:Blank_suedpolarregion.jpg, accessed on 31 October 2025, CC BY-SA 3.0, https://commons.wikimedia.org/w/index.php?curid=285443, accessed on 31 October 2025). (**e**) Distribution of the Ross seal, *Ommatophoca rossii* (By IUCN Red List of Threatened Species, species assessors and the authors of the spatial data., CC BY-SA 3.0, https://commons.wikimedia.org/w/index.php?curid=12198251, accessed on 31 October 2025).

**Figure 6:**
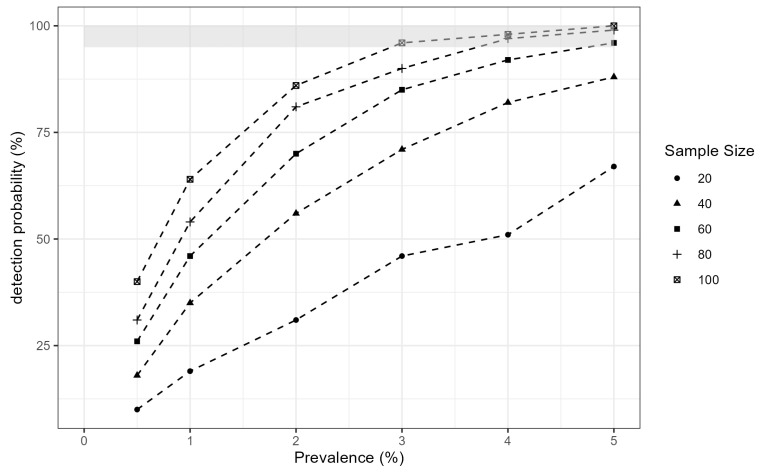
Example of detection probabilities across a range of rabies virus seroprevalence and sample size scenarios for a southern elephant seal breeding colony (N = 6000). The gray band at the top indicates combinations where the detection probability exceeds 95%.

Was the evolving South African situation a unique event? Participants involved in the international conference also considered future wider implications. The semblance of sustained transmission of RABV in South African Cape fur seals (*A. pusillus*) along the southwestern African coast raised concern for Southern Ocean pinniped populations [100,101,102]. This apprehension stemmed from the rare but unpredictable presence of vagrant southern elephant seals (*M. leonina*) and leopard seals (*H. leptonyx*) originating from Antarctica that hauled out along the Cape coast (Figure 3a,b). If exposed, vagrants might potentially introduce RABV into highly susceptible Antarctic ecosystems. Given the overlap of multiple pinniped species in the southern hemisphere in the face of similar contact with local viral reservoirs, the overall lack of rabies coastal surveillance of marine mammals was apparent (Figure 4, Figure 5 and Figure 6).

Moreover, the colonial breeding habits of sub-Antarctic and Antarctic wildlife create dense aggregations of animals and provide suitable conditions for intraspecies as well as interspecies transmission, raising questions about the likelihood of RABV establishment throughout the Southern Ocean marine mammal populations over time (Table 3).

## 4. Discussion

Past incidental findings on pathogens guide current surveillance endeavors, parsed by perceived impacts to agriculture, public health, conservation biology, trade, and economical outlays, etc. From this perspective, expanded lyssavirus surveillance is not a major priority. This is not surprising, considering that a historical problem such as rabies is a neglected viral disease, particularly in lower-and-middle-income countries. Within this space, the detection of RABV in southern African fur seals was only unexpected because the practical concept of host breadth and switching was under appreciated in the realm of both terrestrial and marine mammal species [10]. Superimposed upon this scheme academically, the molecular mechanisms that drive interspecies transmission of RABV and viral adaptation to new reservoir hosts (host shift events) are enigmatic [114]. However, it is evident that host biology, viral genetics, and ecological context each contribute substantially to whether such host switches succeed to become true shifts.

In the case of non-traditional lyssavirus hosts such as pinnipeds, these uncertainties make it especially challenging to predict how a RABV outbreak might progress within abundant dense breeding colonies, compared to terrestrial carnivores, such as canids, raccoons, or skunks [115]. Key parameters governing RABV transmission dynamics in pinnipeds, including the incubation and infectious periods, inter- and intra-species transmission rates, and the clinical course of disease, remain unknown. Consequently, it is difficult to predict how a rabies outbreak would unfold in dense pinniped colonies elsewhere in the southern hemisphere or the Antarctic region. Moreover, is surveillance adequate outside of southern Africa? How likely is it that a host switch would result in a new host shift? Would such a RABV introduction be a dead end, leading to substantial mortality and measurable population declines, or could the virus persist at low prevalence through a metapopulation dynamic across complex pinniped communities? This uncertainty underscores the consideration behind any surveillance and targeted field studies in Southern Ocean seal populations or elsewhere. Enzootic rabies may already be present. In particular, establishing baseline, pre-RABV introduction data is essential for detecting early evidence of viral exposure and transmission. If present, such data are elementary for informing timely, strategic disease control interventions. Any disease surveillance in marine mammals is inherently challenging, requiring consideration of a variety of potential laboratory options for utilization in lyssavirus detection (Table 4).

Mortality events often go undetected because moribund animals make easy prey and carcasses typically sink at sea. Even those that wash ashore may remain unnoticed in remote or inaccessible regions or be scavenged rapidly. Live animal sampling presents further difficulties: while capturing seals is generally more feasible when they haul out on land, restraining large pinnipeds—particularly adult male southern elephant seals, which can exceed two tons—is dangerous and may require chemical immobilization. Moreover, large pinnipeds, such as wild elephant seals, are only accessible during brief periods when they come ashore to breed or molt, further limiting sampling opportunities. While rabies is a highly neurotropic disease, and detection of viral antigens or amplicons is desirable, field collection and storage of CNS tissue is challenging, ideally requiring freshly dead animals (even though detection is possible in degraded samples). Yields may be low. Of all the testing options, serology may be the most ideal, as seen in other Carnivora. Blood sampling has been performed safely in multiple pinniped species. The demonstration of RABV antibodies has been used in both antemortem and postmortem surveys, from bats to carnivores to humans, and other mammals, demonstrating viral activity in a host population [10]. Although minimally invasive techniques for obtaining blood samples have been developed, overall access for routine surveillance remains logistically complex and resource-intensive, making collaborative research critical to marine mammals, rather than de novo activities.

Given these logistical and ecological constraints, if such monitoring is desirable, it is essential to preemptively develop a robust RABV surveillance framework for pinniped subpopulations in the Southern Ocean (Figure 4). Such an approach should explicitly account for the aforementioned challenges: the low likelihood of detecting infected vagrant individuals; low carcass detection rates; limited access to live animals; and the highly seasonal nature of pinniped haul-out behavior. Designing an effective, enhanced surveillance system would require statistically grounded sampling strategies, including calculations of the minimum sample sizes necessary to detect RABV exposure at specified prevalence thresholds and confidence levels. Surveillance data must be interpreted in the context of detection probabilities to avoid false assumptions of disease absence. Ultimately, proactive planning that integrates ecological knowledge, logistical feasibility, and quantitative surveillance design would be vital for early detection of RABV introduction and for any rational, informed international responses, to protect multiple vulnerable Southern Ocean populations, dependent upon findings (Figure 5a–e).

Herein, as one example, we illustrate a relationship between sample size and detection probability to estimate the seroprevalence of RABV antibodies in one vagrant species, a southern elephant seal breeding colony at St Andrews Bay, South Georgia. We initialized an agent-based simulation model with approximately 6000 adult breeding females present on the beaches during the breeding season [36] and a sex ratio of roughly 74 breeding females per alpha male (http://www.eleseal.org/sli/sli_demo.html; accessed on 31 October 2025). Using an iterative approach, we estimated detection probabilities across a range of sample sizes (10–100 animals) and true seroprevalence values (0.5–5%) (Figure 6). A sample size of 100 animals should be sufficient to confidently detect RABV exposure at a true seroprevalence of 3% in a breeding colony (approximately 180 seropositive seals). This assumes that both sampling and the distribution of seropositive individuals are random. At a higher seroprevalence of 5%, a sample size of 60 animals yields a detection probability of approximately 95%. In a real-world surveillance context, this means that if the true seroprevalence of RABV antibodies is 5% and 60 seals are sampled from the breeding colony, there is a 95% probability that at least one seropositive individual will be detected. Conversely, if all 60 samples test seronegative, the 95% confidence interval for the true seroprevalence in the colony will range from 0 to approximately 5%. Moreover, model-based explorations using longitudinal surveillance data can further optimize surveillance efforts and support informed disease control strategies.

Any minimal enhanced regional surveillance begs the question as to whether rabies is already present. If not, how much and how long would it take to detect a low-probability event? Over time, lyssaviruses emerged globally in multiple wildlife reservoirs [121,122,123]. Based upon historical case reports over the past century alone, experimental studies, and ongoing global surveillance, all mammals are believed to be susceptible to RABV [124,125]. This breadth spans the marsupials (e.g., opossums) and diverse placental mammals [126]. Omitting another vertebrate class that includes birds (which are also susceptible) for simplicity, mammalian taxa range within a veritable alphabet soup of possibilities, from armadillos to zebras [127,128,129]. Given the extreme flexibility of these negative-stranded RNA viruses in host switching, emergence in African pinnipeds was somewhat predictable under the ideal epizootiological circumstances.

The implications apply to other regions, including the Arctic and the Tropical realms. Contact with pinnipeds occurs with infected vampire bats and mesocarnivores along the New World coasts (e.g., Peru, Brazil) due to predator–prey dynamics. Such engagement is perhaps a less significant concern in Australia, with the only risk being exposure to lyssavirus-infected flying foxes, unless infected vagrants were to arrive and introduce RABV into resident coastal fur seals. Although human and equine cases have occurred, no Australian lyssavirus spillover infections from bats have been reported to carnivores thus far. Given evolutionary relationships between pinnipeds and carnivores such as canids, the probability of a spillover infection resulting in a host shift event may be higher with RABV of more prospective caniform ancestry and adaptations, than from jumps by genetically more distant bats to carnivores in the same time and space [123]. Perturbations from climate change and other anthropogenic factors make predictions difficult. At a minimum, based upon this southern African scenario, opportunities for enhanced surveillance, collaborative research, shared samples, modeling attempts, and future approaches for disease mitigation abound, with extension from the biodiversity of lyssaviruses and hosts, based upon current events [130].

Our focused review of pinniped rabies demonstrated how the paucity of information impacts scientific opinion and actions related to global surveillance, public health recommendations, and management applicable to conservation biology. This emerging event of African seal rabies confirms that, similar to terrestrial mammals, pinnipeds are susceptible to RABV infection, as expected from the original 20th century sole case report in a European seal. However, rather than a single, incidental finding of a spillover infection from a rabid jackal, this is an unprecedented, ongoing outbreak in the South African Cape fur seal population, now extended along the coast of Namibia [97,98,99,100,151]. As such, it is the first known instance of RABV spreading among marine mammals.

Prior to this outbreak, it was believed that rabies in seals was exceedingly rare, with only one other confirmed single finding reported in a ringed seal in Norway during the early 1980s, which apparently was a terminal ‘dead-end’ infection and did not result in apparent further cases [43]. Similar to other rabid Carnivora, infected seals exhibit highly abnormal signs, including paresis, paralysis, and unprovoked aggressive behavior, leading to attacks on humans and pets in coastal areas. This unusual aggression was initially attributed to other factors.

Authorities have warned the local public, including tourists, swimmers and surfers, to be extremely cautious and to keep their distance from seals. No human cases of rabies contracted from a seal have been confirmed so far, but scores of people have been bitten since the retrospective case detected in 2022 [96,100,130,151].

Animal management to date has been conservative. Population reduction of seals was not recommended. Local authorities are managing the outbreak through monitoring, collection, and testing of suspect animals, and targeted vaccination of habituated seals and vagrants in urban harbors or rehabilitation centers. Historically, such parenteral vaccination of mesocarnivores elsewhere was practiced on a diverse spectrum of terrestrial species, including raccoons and skunks. The Two Oceans Aquarium in Cape Town is leading a ‘novel vaccination trial’ on specific, high-risk animals. Seals are tagged after vaccination to track their movements and to monitor the safety and effectiveness of the inactivated rabies vaccine [100]. It is uncertain how effective the existing rabies vaccines for terrestrial taxa will be in marine mammals, as no efficacy trials had been conducted on seals before this outbreak. However, modern commercial rabies vaccines are pure, potent, safe, and highly efficacious by the parenteral route in a variety of domestic and wild mammals [131]. Hence, parenteral vaccination should be safe and immunogenic. Such a scheme is considered a measure to protect specific seals, prevent RABV transmission to humans, and minimize further spread to other species. A commercial inactivated vaccine can be administered parenterally to an individual seal [103,104], but mass vaccination of the entire population is not considered practical due to the large number of animals (i.e., 2 million Cape fur seals alone) and the vast coastlines they inhabit from South Africa to Angola [100,130].

Traditional use of ORV used for terrestrial wildlife, such as foxes and raccoons, may be ineffective for wild seals, because pinnipeds typically only consume live seafood, making such typical baiting methods less useful (Table 3) [105]. Hence, the goal of the targeted parenteral vaccination program is to try and interrupt viral transmission and reduce the risks of overall spread, including to other vulnerable seal species.

**Figure 7:**
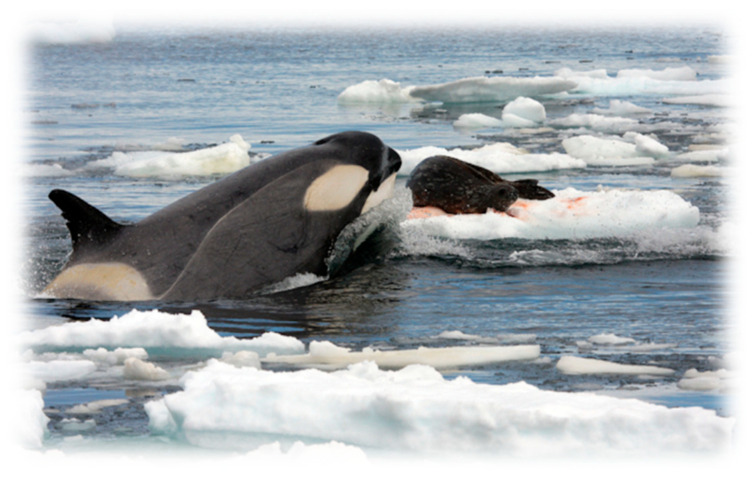
An orca hunting a seal—an opportunity for viral spread among marine mammals? (Robert Pitman—NSF Office of Polar Programs https://commons.wikimedia.org/w/index.php?curid=12655370, accessed on 31 October 2025).

Concerns were expressed about the possibility of the virus spreading broadly to other marine mammals (Figure 7) [100,132]. Given the extreme host breadth, it is possible for any mammal to contract rabies after bite or non-bite exposure via RABV-laden saliva, but the risk of viral transmission from an infected seal to a non-pinniped, such as an orca (*Orcinus orca*), is considered extremely unlikely in the marine environment. Cetaceans have very different behavior patterns from pinnipeds and are fully aquatic [133]. Pinnipeds spend time both in water and on land in dense colonies, where the current outbreak is spreading through routine behavior, such as conspecific fighting and biting. Moreover, RABV is primarily spread through the saliva of an infected animal via a bite or deep scratch. A seal would have to bite an attacking orca to transmit RABV. This is improbable due to the size differences (i.e., comparatively, most whales are massive predators such as orcas, which rarely interact with seals in a way that would provide ideal opportunities for defensive bites) and the difficulty of a seal biting through a cetacean’s thick blubber. While viral excretion quantification is lacking, pinnipeds do have salivary glands and are capable of a productive infection, based upon the current epizootic [100,134,135]. In contrast, whales and dolphins have vestigial salivary glands, likely making a primary productive viral infection largely ineffective in the ocean [136]. Environmental contamination is not a major concern. Lyssaviruses are membrane-bound and comparatively fragile. As such, given dilution effects in the massive volume of the open ocean, widespread waterborne transmission is not envisioned. To date, there has never been a recorded case of rabies in a whale, dolphin, or porpoise. The greater concern centers on viral spread to humans, dogs or other seal species (e.g., vagrant elephant or leopard seals) that may ‘haul out’ in the same areas as Cape fur seals. If an orca were to be infected with RABV, the disease would almost certainly be fatal once signs appeared, leading to incoordination, difficulty of swimming and breathing skills, disruption of echolocation, and compromise of complex social coordination for hunting, navigation, consequent confusion and an inability to function within its pod. Such neurological distress and loss of motor skills would likely lead an orca or other cetacean to become highly disoriented, potentially causing it to beach itself, a common occurrence in distressed marine mammals, or to become prey to other marine consumers.

Many unknowns are obvious related to rabies in pinnipeds. It is highly unlikely that experimental pathobiological studies, such as those performed in terrestrial mesocarnivores, will be forthcoming [4]. A variety of approaches should be generated a priori to predict the likelihood of additional host shifts to pinnipeds and to ascertain the ideal means of any enhanced laboratory-based surveillance of at-risk species in remote locations throughout the Southern Ocean. Such modeling would be useful to integrate a best practices scheme for any holistic management to minimize any broader impacts to conservation biology [137].

Antarctica is a natural scientific reserve for the promotion of scientific endeavors. This includes the obvious health of its fauna and those that study the native inhabitants. To date, this fifth-largest continent and the numerous islands of the Southern Ocean are supposedly ‘rabies-free’, but this international narrative violates elementary considerations due to a lack of any laboratory-based monitoring in the face of predictive risks [138]. An extensive focus on rabies in Cape fur seals serves as a model for introspection elsewhere and a nidus for potential introduction to other locales and species. Besides the socio-political neglect of canine rabies elimination for human health benefits, the scientific community’s overall lack of awareness of lyssavirus evolutionary plasticity and potential for emergence to impact threatened or endangered pinniped and other wildlife populations may limit collaborative global opportunities for surveillance and management in advance of conservation threats with objectivity, transparency, and technology, because ‘…qui non quaerit, non invenit…’ [139]. Nearly a century of introspection upon the evolution and biological success of these negative-stranded RNA viruses projects the obvious risks and the dangers of ignorance [140,141,142,143,144,145,146,147,148,149,150,151].

## Figures and Tables

**Table 1:** Examples of lyssavirus detection among individuals, representative of diverse families of Carnivora, as evidence of incidental host switches from infected reservoirs to another species, typified *primarily* as dead-end infections [14].

Family	Reference
Nandiniidae (e.g., Palm civet, *Paradoxurus zeylonensis*)	[15]
Felidae (e.g., cougar, *Puma concolor*)	[16]
Viverridae (e.g., African civet, *Civettictis civetta*)	[17]
Hyaenidae (e.g., striped hyena, *Hyaena hyena*)	[18]
Herpestidae (e.g., meerkat, *Suricata suricatta)*	[19]
Canidae (e.g., African wild dog, *Lycaon pictus*)	[20]
Ursidae (e.g., black bear, *Ursus americanus*)	[21]
Mephitidae (e.g., Yucatan spotted skunk, *Spilogale yucatanensis*)	[22]
Ailuridae (e.g., red panda, *Ailurus fulgens*)	[23]
Procyonidae (e.g., kinkajou, *Potus flavus*)	[24]
Mustelidae (e.g., yellow-throated marten, *Martes flavigula chrysospila*)	[25]
Various (2851 cases in 17 different Carnivora species over a 40-year period)	[26]

**Table 2:** Selected examples of rabies virus host shift events in the Carnivora (note the predominance of canid variants), which may perpetuate for decades or longer.

New Hosts	Viral Variant	Location	Reference
Red fox (*Vulpes vulpes*)	Arctic Fox	Canada	[142]
Striped skunk (*Mephitis mephitis*)	Arctic Fox	Canada	[143]
Grey fox (*Urocyon cinereoargenteus*)	California Skunk	USA	[144]
Striped skunk (*Mephitis mephitis*)	Cosmopolitan Dog	USA	[145]
Coyote (*Canis latrans*)	Mexican Domestic Dog	USA	[146]
Small Indian mongoose (*Urva auropunctata)*	Cosmopolitan Dog	Cuba	[147]
Crab-eating fox (*Cerdocyon thous*)	Dog-associated	Brazil	[148]
Ferret badger (*Melogale moschata*)	Dog-associated	China	[149]
Black-backed jackal (*Canis mesomelas*) and Bat-eared fox (*Otocyon megalotis*).	Dog-associated	South Africa	[55]
Aardwolves (*Proteles cristatus*)	Dog-associated	South Africa	[55]
Yellow mongoose (*Cynictis penicillata*)	Canine Africa-3	Southern Africa	[123]

**Table 3:** General characteristics of selected pinniped (seal) species found in the southern hemisphere that are at potential risk of rabies virus exposure, based upon occasional host switching via the degree of interaction with infected terrestrial counterparts [27].

Pinniped	Distribution	Estimated Population	Comment	Reference
Cape fur seal (*Arctocephalus pusillus*)	Non-migratory residents of coastal Angola, Namibia, South Africa, and parts of Australia (subspecies *A. p. doriferus*)	~1,000,000–2,000,000	Frequent contact with domestic animals (e.g., dogs and cats), other wildlife (e.g., jackals), and humans while on land	[28]
Southern elephant seal (*Mirounga leonina*)	Migrate from small islands in the Southern Ocean to Antarctica	~300,000–600,000	As the largest-bodied carnivores, they are more solitary vagrants, except during the breeding season	[29]
Subantarctic fur seal (*Arctocephalus tropicalis*)	Breed on small islands of the Southern Ocean	~200,000–300,000	Range overlaps with Antarctic seal (*Arctocephalus gazella*), and occasional vagrants to other continental coasts	[30]
Leopard seal (*Hydrurga leptonyx*)	Predators of the open Southern Ocean and occasional coastal continental vagrants	~18,000–20,000	Adults feed upon fish, penguins, and other seals and are occasional vagrants to other continental coasts	[31]
Weddell seal (*Leptonychotes weddellii*)	Circumpolar pack ice of Antarctica	~300,000	Deep-diving, opportunistic, non-migratory seals adapted to ‘fast’ or connected ice, with much less opportunity for viral exposure, but non-adults may be prey of leopard seals	[32]
Ross seal (*Ommatophoca rossii*)	Circumpolar pack ice of Antarctica	Uncommon, smallest, and least well known of the Antarctic seals	Primarily feed on fish, squid or other invertebrates, and may experience predation by leopard seals and killer whales	[14,27]
Crabeater seal (*Lobodon carcinophaga*)	Typically found on floating sea ice fragments of the Southern ocean, and as occasional vagrants on the coasts of South Africa, Australia, New Zealand, and Brazil	Most abundant seal, estimated at several million, but difficult to enumerate because they do not form mainland colonies	Adapted to consume krill, and occasional vagrants to the continental coasts	[33]

**Table 4:** Potential diagnostic tools for enhanced surveillance of rabies in pinnipeds.

Method	Comment	Reference
Histology	Observation of microscopic changes in CNS	[116]
Antigens	DFAT/RIT/LFA of CNS	[117]
Nucleic acids	RTPCR of CNS, saliva	[118]
Serology	Antibodies via ELISA and neutralization (e.g., FAVN, RFFIT)	[119]
Environmental sampling	Need specific targets to bias success	[120]

## Data Availability

No new data were created or analyzed in this study. Data sharing is not applicable to this article.

## Abbreviations

The following abbreviations are used in this manuscript:

DFAT	Direct fluorescent antibody test.
ELISA	Enzyme-linked immunosorbent assay.
FAVN	Fluorescent antibody virus neutralization test.
IHC	Immunohistochemistry.
LFA	Linear flow assay.
ORV	Oral rabies vaccination.
PEP	Post-exposure prophylaxis.
PrEP	Pre-exposure prophylaxis.
RABV	Rabies virus.
RFFIT	Rapid fluorescent focus inhibition test.
RIT	Rapid immunohistochemistry test.
